# Using ecotourism boats for estimating the abundance of a bottlenose dolphin population in south-eastern Australia

**DOI:** 10.1371/journal.pone.0289592

**Published:** 2023-08-04

**Authors:** Paola Lacetera, Suzanne J. Mason, Paul Tixier, John P. Y. Arnould

**Affiliations:** 1 School of Life and Environmental Sciences, Faculty of Science, Engineering and Built Environment, Deakin University, Burwood, Victoria, Australia; 2 Faculty of Science, Krijgslaan, Gent University, Gent, Belgium; 3 Cetacean Science Connections, Forest Hill, Victoria, Australia; 4 MARBEC, Université de Montpellier-CNRS-IFREMER-IRD, Sète, France; MARE – Marine and Environmental Sciences Centre, PORTUGAL

## Abstract

It is challenging to collect robust, long-term datasets to properly monitor the viability and social structure of large, long-lived animals, especially marine mammals. The present study used a unique long-term dataset to investigate the population parameters and social structure of a poorly studied population of bottlenose dolphins (*Tursiops* sp.) in southern Port Phillip Bay, south-eastern Australia. Photo-identification images have been collected between 2012–2022 both opportunistically and following a protocol by patrons, staff, and volunteers of ecotourism companies using their vessels as platforms. The resulting large dataset was available to be processed through the online platform *Flukebook* and used in capture recapture models to estimate abundance and demographic parameters. In addition, the social structure of the population and the reproductive parameters were investigated. The marked adult population abundance (45.2 ± 2.7 individuals) was found to be stable over the last decade and the calving rate ranged between 0.06–0.19 new calves per identified individuals per year, while the inter-birth interval was 3.7 ± 0.8 years. Social analysis suggested the population has a fission-fusion structure with no apparent clusters. The stability of the population over the study period suggests no deleterious effect of anthropogenic or environmental factors during the last decade. This study is the outcome of the effort of the ecotourism organisations and the results obtained, along with their similarity to those of other dolphin populations worldwide, highlight the importance of such data sources for long-term information that would otherwise be too expensive or logistically difficult to obtain.

## Introduction

A deep understanding of large-scale variation, short- and long-term fluctuations, ecosystem regulations, uncommon events and disturbances, and the effect of anthropogenic pressure on ecosystems can be obtained only through long-term studies [1–3]. While their importance is clear, long-term studies are not common for many reasons, ranging from insufficient funding to inefficient communication [4]. Some of these challenges can be overcome by involving citizen science and third parties in collecting data, which has proven successful in obtaining reliable results in a variety of settings [5, 6].

In species-driven research, particularly on long-lived species like large mammals, demographic and reproductive parameters such as survival, population size, recruitment, and population growth rate are the first parameters to be investigated to assess the status of the population [7–10]. Long-term monitoring of the fitness of a population is fundamental as it is affected by slow-changing environmental factors [11]. For instance, the social structure of a social species reflects the trade-off between the costs and benefits of being alone or part of a group based on resource type and allocation [12, 13], intra-specific competition, and predation [14, 15]. Both environmental factors and the social structure of a population affect its demographic and reproductive parameters and, consequently, its long-term viability [7, 16–18].

Demographic and reproductive parameters can be obtained using photo-identification, which is one of the least invasive and least expensive techniques for collecting long-term data relying on unique, long-lasting natural markings (i.e. skin patterns, lesions, scars) to identify individuals [19–21]. For instance, a study on the communities of African elephants (*Loxodonta africana*) in Tanzania used photo-identification, together with other techniques, to assess the long-term impacts of poaching on fitness by looking at social structure, stress, and reproductive parameters [22]. However, obtaining photo-identification of large mammals over long periods can be logistically difficult. Automated techniques such as camera traps have been used in some instances [23–28] but require individuals to regularly past stationary cameras [29]. Collecting photo-identification becomes even more challenging when highly mobile marine species are involved, as they cover vast areas and spend most of their life submerged [19, 30].

Consequently, due to the financial costs, logistical constraints and time limitations, photo-identification data collection is increasingly relying on citizen scientists and third parties to develop robust datasets on marine species. For example, such non-traditional data sources have been used in studies of whale sharks (*Rhincodon typus*) [31], manta rays (*Mobula alfredi*) [32], and cetaceans [33, 34]. Once an accessible and reliable sampling method to collect data of sufficient quality has been developed, long-term, robust datasets can be accumulated and reliable population monitoring established.

Some of the most studied cetaceans are the bottlenose dolphins (*Tursiops* spp.) [35], which are long-lived, highly social species with a worldwide distribution inhabiting coastal and offshore waters [36]. They are encountered in groups defined as individuals close together engaging in the same behaviour or travelling in a similar direction [37–39]. Since they usually live in fission-fusion societies, the combinations of individuals forming a given group change frequently. They also show preferred long-term associations based on sex, age, and feeding or hunting techniques which lead to the formation of clusters [40–44]. Hence, a population, defined as a group of individuals of the same species living in the same area and interbreeding [45], can be composed of several social clusters which can be link to one another even by one individual [44]. The consequences of environmental changes, direct and indirect impacts of anthropogenic activities, infectious diseases, and pollution, could affect their fitness and long-term viability [46–48]. Consequently, collecting data to assess the population status is fundamental and, since individuals can be identified from unique features of their dorsal fin, they are suitable for long-term studies using photo-identification [49]. Indeed, individual bottlenose dolphins have been monitored for years using photo-ID techniques in studies in Scotland [50, 51] and in Australia [52], and many other cetaceans worldwide [53, 54].

Southern Port Phillip Bay, south-eastern Australia, is home to a small, poorly-studied population of bottlenose dolphins [55, 56], which has been recently proposed as a new species, the Burrunan dolphin (*Tursiops australis*) [57, 58]. Since it is one of only two known populations of *T*. *australis* (the other being resident in the Gippsland Lakes [57, 58]), is small, shows female philopatry, and is frequently in contact with many anthropogenic activities [59], it has recently been listed as Critically Endangered under the State of Victoria’s Flora and Fauna Guarantee Act 1988 [60]. However, as the new species has not been recognised by the Society for Marine Mammalogy [61], it will be referred to hereafter as bottlenose dolphin (*Tursiops* sp.).

The dolphins of southern Port Phillip Bay are the focus of four permitted “dolphin watching and swim with dolphins” ecotours, involving frequent approaches and encounters by tour boats, which, has led to concerns being raised about the health and safety of the population [55, 62, 63]. Encounters are defined as the period of time during which the ecotour vessels are interacting with the dolphins [64]. In addition, south-eastern Australia is one of the fastest warming oceanic regions in the world and the anticipated climate change impacts could greatly affect marine predators in the region [65–67]. Furthermore, such effects could be amplified for populations being resident in shallow, semi-enclosed areas like Port Phillip Bay [63].

Research on bottlenose dolphins in Port Phillip Bay has been hampered by its large area (ca. 1900 km^2^) and the logistical and financial constraints this poses [68]. No long-term peer-reviewed studies providing abundance estimates nor information on the social organization are available for the population despite these aspects being fundamental to developing appropriate management protocols. In particular, the way habitat and resources are used by individuals may vary and this can result in heterogenous responses to pressures and conservation measures [69, 70]. With ever-increasing pressures on environmental management budgets, new economically viable methods of data collection are required [34, 71, 72]. Correspondently, the four ecotourism boat charter companies active in the region present such an opportunity. This is especially so considering the reliable frequency and effort with which they can obtain suitable data [73].

The aims of this study, therefore, were to determine the: 1) current abundance; 2) long-term trend; and 3) social structure and reproductive parameters of bottlenose dolphins in southern Port Phillip Bay, using ecotour boats as platforms for collecting photo-identification data.

## Methods

### Study area, data collection and processing

**Fig 1:**
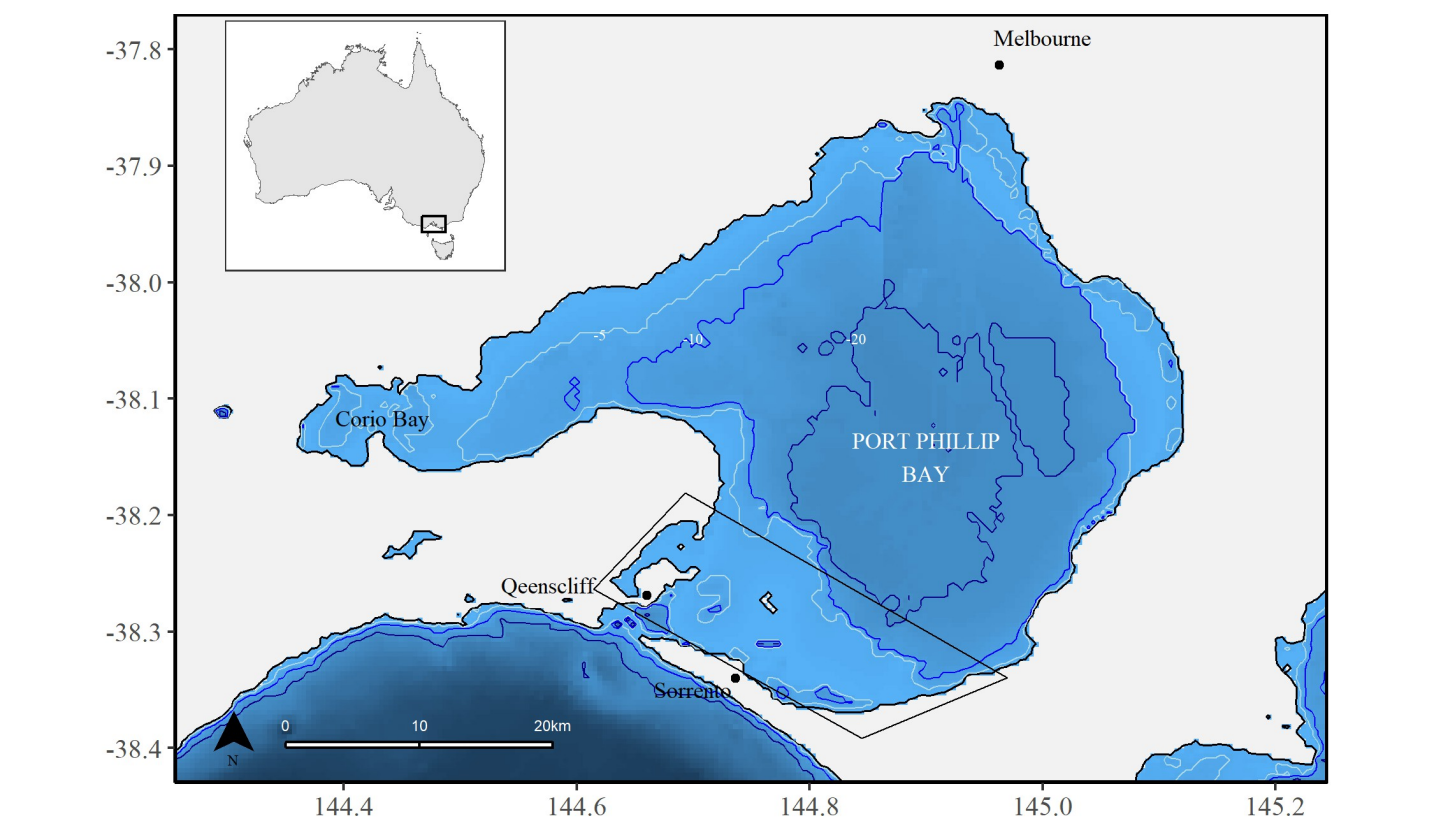
Map of Port Phillip Bay, south-eastern Australia. The study area of ca. 200 km^2^ is identified by the polygon. The map was obtained in the R statistical environment [80] using RStudio (version 2022.2.1.461), the ozmaps package [81], and GEBCO bathymetry data (GEBCO Gridded Bathymetry Data) [82].

Port Phillip Bay is a large, shallow (maximum depth of 24 m), semi-enclosed bay on the northern coast of Bass Strait, south-eastern Australia (Fig 1). The entrance at the southern end of the bay, known as “the Rip”, is ca. 3 km wide [74, 75]. This narrow entrance greatly influences the bay by limiting the exchange of water with Bass Strait and affecting its hydrodynamics [68, 76]. Consequently, it is also thought to restrict species movements, with invasive species introduced mostly through maritime traffic [77]. Even marine mammals normally non-resident in semi-enclosed bays, such as common dolphins (*Delphinus delphis*), have been observed being resident in Port Phillip Bay [78, 79].

The southern end of Port Phillip Bay is the focus of four “dolphin watching and swim with dolphins” commercial tour operators [83, 84]: *Moonraker Dolphin Swims*, *Polperro Dolphin Swims*, and *WaterMaarq Seal and Dolphin Swims* which depart from Sorrento, and *Sea All Dolphin Swims*, which departs from Queenscliff (Fig 1). These companies meet the criteria for ecotourism as defined by [85–87]. Images were collected by patrons and staff between October 2012 –April 2022 both opportunistically and following a protocol on-board the *Moonraker Dolphin Swims*, *Polperro Dolphin Swims*, and *Sea All Dolphin Swims* vessels and were obtained with permission for the present study. The data collection area was restricted by the operational limits of these companies (ca. 200 km^2^) (Fig 1), and the data collection period was limited to their activities which run from the austral spring to autumn (October–April). A variety of different digital SLR camera models were used throughout the study period with focal lengths ranging between 80–400 mm. The platforms from which photographs were taken were on average 3 m above sea level, and sea-state conditions during the data collection were never greater than Beaufort Sea State 3.

Data for this project were collected under whale (and dolphin) swim tour operator tour permits issued by the Victorian Department of Environment, Land, Water, and Planning for the limited permit area of Port Phillip. At no point during the study were tour operators requested to operate outside of their permit conditions to collect data.

Using the methods of Urian et al [88], each individual representation of a dolphin in a digital image was graded by assessing five photographic characteristics assigning one of the available predefined absolute values: focus (2 = excellent, 4 = moderate, 9 = poor), contrast (1 = ideal, 3 = excessive/minimal), angle (1 = perpendicular, 2 = slight, 8 = oblique), partial or full fin (1 = full fin, 8 = partial). and the proportion of the frame filled by the fin (1 = greater than 5%, 5 = less than 1%). The sum of the five scores would result in *poor* (>12), *average* (10–12), or *excellent* (6–9) picture quality. In addition, further following Urian et al [88], the distinctiveness of the dorsal fin was scored based on how many marks, notches, and scars were present, and their size. A very distinct fin would be scored as D1 (more than two major distinctive features), an average one as D2 (one or two major distinctive features), and a clean fin as D3. The side of the fin (left or right) was noted as well as other metadata such as location, date, time, photographer, and vessel.

To identify individual dolphins and create the catalogue for Port Phillip Bay bottlenose dolphins, all the *excellent* and *average* quality images were uploaded to the online cetacean photo-identification portal *Flukebook*. *Flukebook* (www.flukebook.org) is one of the many available *WildMe* platforms that uses the *Wildbook* system to rapidly analyse populations through machine learning and computer vision [89]. Bottlenose dolphins images are analysed with two different algorithms [90]: CurvRanch v2 [91] and finFindR [92]. The first matches curvatures of the trailing edge of dorsal fins, considering the weight of the section of the curvatures so that the identification can be successfully carried out even when the animal is deformed by changes in the pose or angle [91]. The second matches curvatures as well, but the machine learning process is mostly used to find the cetacean body, identify its fin, extract the edge, and create a feature matrix for the fin [92]. Both these algorithms are automatically used by Flukebook to find the best fin match, which then must be manually selected. With time, the machine learning process improves and the options available to choose the correct fin match from become more accurate.

In *Flukebook*, the species, date, and time of each image were specified as well as a predefined location ID, which was Port Phillip Bay. Every time *Flukebook* found a fin in an image, it ran the previously cited algorithms to identify the fin, created a matrix of fin features, and compared it to the others in the catalogue based on location. The results of the matching process were returned as a list of fins ranked by the highest score of similarity. The correct match was then manually selected, and a unique identifier was given to the newly identified dolphins.

Data collection years were defined by the year in which the spring season occurred. Data processing and exploration were conducted in the R statistical environment [80] using RStudio (version 2022.2.1.461).

### Capture recapture models

The capture recapture (CR) method was used to estimate population abundance and demographic parameters of bottlenose dolphins in southern Port Phillip Bay by modelling capture and survival probabilities of individuals [93–95]. Since the movements of the bottlenose dolphins in southern Port Phillip Bay are not well known, and the study site is relatively small, the robust design (RD) model was chosen as it accounts for temporary emigration, and primary and secondary sampling occasions are defined [96, 97]. Between primary occasions (years), the population is assumed open and apparent survival and temporary emigrations can be estimated [98]. Between secondary occasions (months), the population is assumed closed and capture probabilities and population abundance can be estimated [99]. In the present study, the RD primary and secondary occasions were defined as follows: the whole sampling period goes from 2012–2021, for a total of 10 primary occasions (years) and up to 5 secondary occasions (months between November–March). However, due to a shift in effort caused by the COVID-19 pandemic, data in year 2020 was only collected between January–April. Consequently, April was included as a secondary occasion in that year. Recaptures in a secondary occasion (month) were obtained by pooling daily recaptures of each individual within each month [100]. Only adult dolphins were considered in this analysis. Calves and juveniles, identified as being <80% of adult body size [101–103], were excluded by comparing sizes through photo-identifications (photo-IDs). Furthermore, calves and juveniles can be recognised by being usually less heavily marked and by their appearance [95]. Records of calves and juveniles were kept and used for the reproductive analysis.

In order for RD models to be valid, they must meet several assumptions:

1. *Individual marks are correctly recognised and are not lost over time–*only *excellent* and *average* quality photographs were used to identify dolphins; the identification process was carried out in *Flukebook*, which uses artificial intelligence to limit errors; the marks used for the identification do not allow for misidentifications as they are low in loss and gain rates [104] (i.e. main cuts and notches on trailing edge of the fin); in the model only D1 and D2 individuals were included;
2. *Sampling sessions should be of a shorter duration than the total sampling period*–for each sampling year, only data collected between November–March was considered, so that there was a maximum of 5 months’ worth of images per year (i.e. up to 5 secondary occasions within a primary occasion);
3. *The population is closed within primary periods*–additions and deletions within primary periods (years) could be considered negligible as bottlenose dolphins have high survival rates and are long-lived animals [105] and each primary period was made up by maximum 5 months; even if calves were identified, they were excluded from the analysis; the closure assumption was also checked through the Close Test Program [99, 106];
4. *Equal survival and capture probability among individuals*–*R2ucare* package [107] was used to test these assumptions with the overall test for the Goodness of Fit.

The parameters of the models are defined as follows: *S* is the survival probability (apparent survival) which is the probability of surviving and staying in the study area and it results from the multiplication of true survival and site fidelity; *p* is the capture probability; *Y”* is the probability an individual temporarily emigrates from the study area, given it was present and alive in the previous sampling occasion; *Y’* is the probability an individual temporarily emigrates from the study area, given it was a temporary emigrant in the previous sampling occasion; and *N* is the population size of the marked individuals in the study area.

All the models were run in R using the *Rmark* package [108]. Three temporary emigration scenarios were considered:

1. No temporary emigration, where *Y”* = *Y’* = 0;
2. Random temporary emigration, where *Y”* = *Y’*;
3. Markovian temporary emigration, where *Y”* ≠ *Y’*.

When temporary emigration is considered random, the probability of an individual being present in the study area at the sampling occasion is not dependent on its presence during the previous sampling occasion. Instead, the Markovian approach assumes dependency on its previous presence in the study area [96]. For each of the three scenarios, *p* was set either constant or could vary with time between secondary or primary occasions, or both. *S* was set to either constant or time dependent in primary occasions. The best-fitting model was chosen based on the Akaike’s Information Criterion (AIC).

### Social network analysis and reproductive parameters

To investigate the social organisation of the population of bottlenose dolphins in southern Port Phillip Bay, a social network analysis was conducted in SOCPROG 2.4 [109]. Daily sampling periods were chosen and associations during a sampling period were defined as dolphins in the same group. No information about encounter nor group number was available. Hence, groups were defined based on the time of the photographs. A gap of >15 min between two photographs was considered as a newly encountered group. This threshold was chosen as tour boats are licensed to spend a maximum of 20 min swimming with dolphins and a maximum of 20 min within 100 m from them. Even though these limitations are not always followed [56], the threshold was deemed appropriate.

The basis of a social network analysis is the association between two dolphins, which comes with the associated Standard Error (SE). Here, associations were estimated using the Half-Weight Index (HWI), which is defined as follows:

$$HWI=\frac{X}{X+1/2(Ya+Yb)}$$

- *X* is the number of groups including both dolphins *a* and *b*
- *Ya* is the number of groups only including dolphin *a*
- *Yb* is the number of groups only including dolphin *b*

The index ranges between 0–1, 0 being two dolphins (*a* and *b*) never associated, and 1 always associated. It is the most commonly used index, especially in studies of bottlenose dolphins, and it is the least biased when not all the individuals can be identified. Associations greater than 0.65 were considered strong [110, 111]. The presence of preferred associations was assessed through a Monte Carlo permutation test (*permute groups within samples*) advised for long-term studies and implemented in the SOCPROG software [110, 111] following the modified methods of Bejder et al. [112]. To ensure the accuracy of the *P* value, the observed matrix of association indices was permutated from 1000–4000 replicates until the *P* value stabilised. If the obtained SD (standard deviation) of the real association indices is significantly high, long-term preferred associations are detectable, while a significantly low mean suggests short-term preferred associations [110, 111].

Understanding whether a population is divided into social clusters is fundamental as different clusters could use space and resources differently and, consequently, respond distinctively to environmental changes and management measures [69, 70]. Hence, the social structure was investigated using the hierarchical cluster analysis with the four available methods: single-linkage, complete-linkage, average-linkage, and Ward’s-linkage. The most accurate one was chosen based on the highest cophenetic correlation coefficient (CCC). A reliable dendrogram is obtained when the CCC is higher than 0.8 [110, 111]. Also, a network diagram was produced to display the associations without assuming a hierarchically organised social structure. A modularity metric (Q) was calculated which, if greater than 0.3, would suggest a good representation of the division into clusters [110, 111]. It is also important to know the role that each individual dolphin plays within the society [41]. Hence, the network analysis statistics were obtained, together with their SE with a Bootstrap method of 1000 replicates. They were defined as follows: strength reflects the ability of an individual to form strong associations; the eigenvector centrality measures how well an individual relates to conspecifics and how well they are connected; reach assesses indirect associations; the clustering coefficient indicates how well the individuals associated with a focal dolphin are themselves associated; affinity measures the strength of its associates [110, 111].

Lastly, a temporal analysis was carried out to assess the stability of associations over time through lagged association rates (LAR). Through this analysis, it is possible to confirm that the society shows fission-fusion characteristics [110]. The LAR is the probability that if dolphin *a* and *b* are associated at time *t*, they will associate again at time *t* + *d*, *d* being a specified time lag. Estimates of precisions of the LAR averaged over all individuals were obtained using a jack-knife procedure [110, 111]. As described by Whitehead [110, 111], exponential decay models were fitted to include the following social elements differently combined: rapid disassociations, preferred companions, and casual acquaintances. The most parsimonious model to describe the temporal dynamics of the social structure was obtained using AIC selection [110, 111].

Calf data were used to calculate some reproductive parameters which can suggest changes in population health. When calves were identified alongside an adult, they were classified according to size as having been born that year (*newborn*) or being at least 1 year old (*olde*r). The relationship between the number of calves seen per year and the effort (days in which photographs were obtained) was assessed through a GLM. The commonly used reproductive parameters of the inter-birth interval (IBI) and calving rate (*cr*) were assessed [101, 113–115]. To obtain both, the older calves were backdated by one year to estimate the number of calves born in a given year. The IBI was calculated from each adult dolphin seen with multiple calves throughout the study period by counting the number of years between new calf captures, and the mean across individuals was obtained. The *cr* was calculated for each year by dividing the number of calves born by the number of identified adults in each year. Unless differently specified, in this study, *P* < 0.05 was chosen as statistically significant and the mean values are reported with their SE as a measure of accuracy.

## Results

### Recaptures

**Fig 2:**
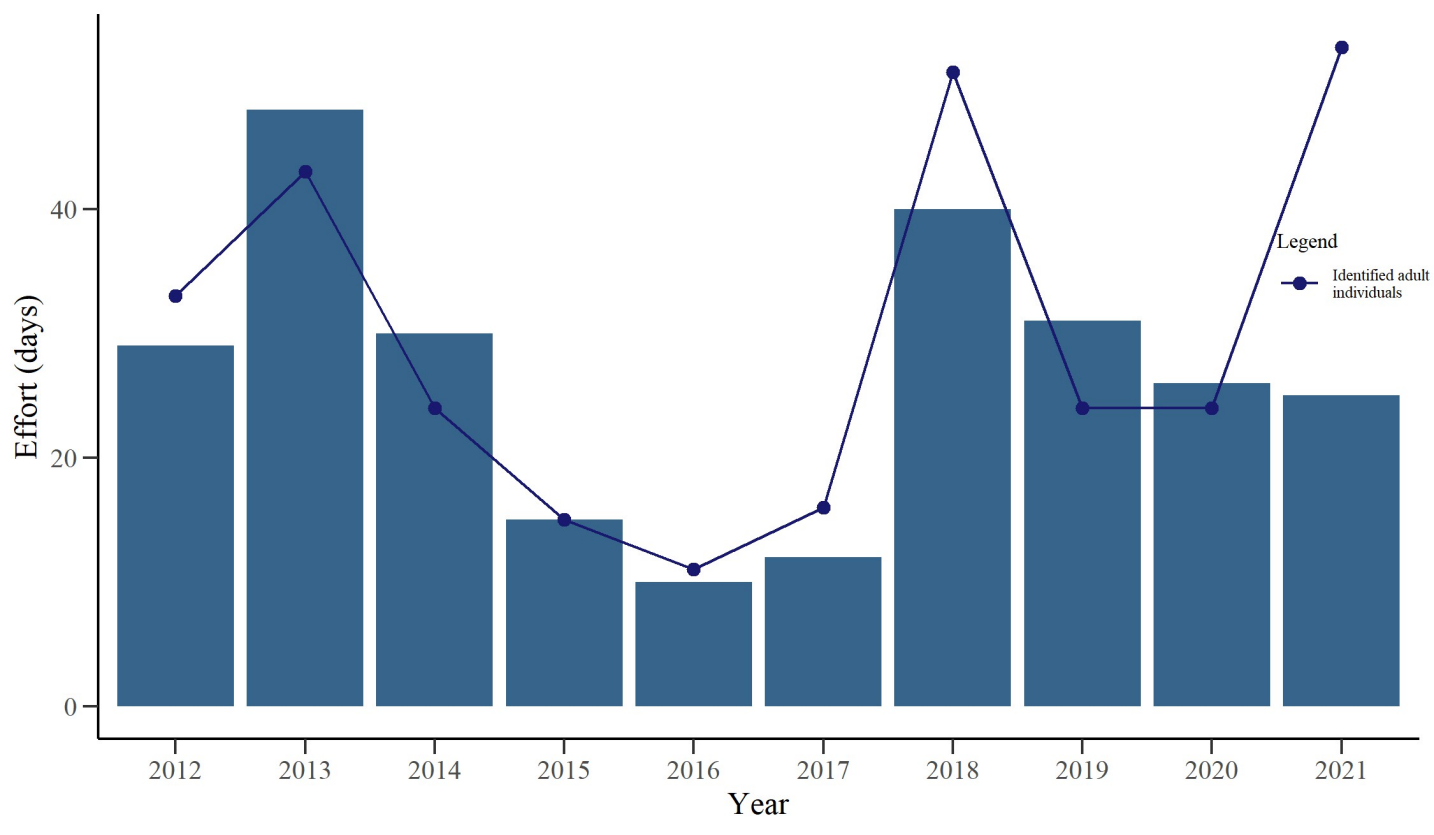
Identified adult individuals and data collection effort. Bar plot showing the effort as the number of days in which digital images were collected each year of the study and how many adult bottlenose dolphins (*Tursiops* sp.) were identified in southern Port Phillip Bay, south-eastern Australia, each year.

**Fig 3:**
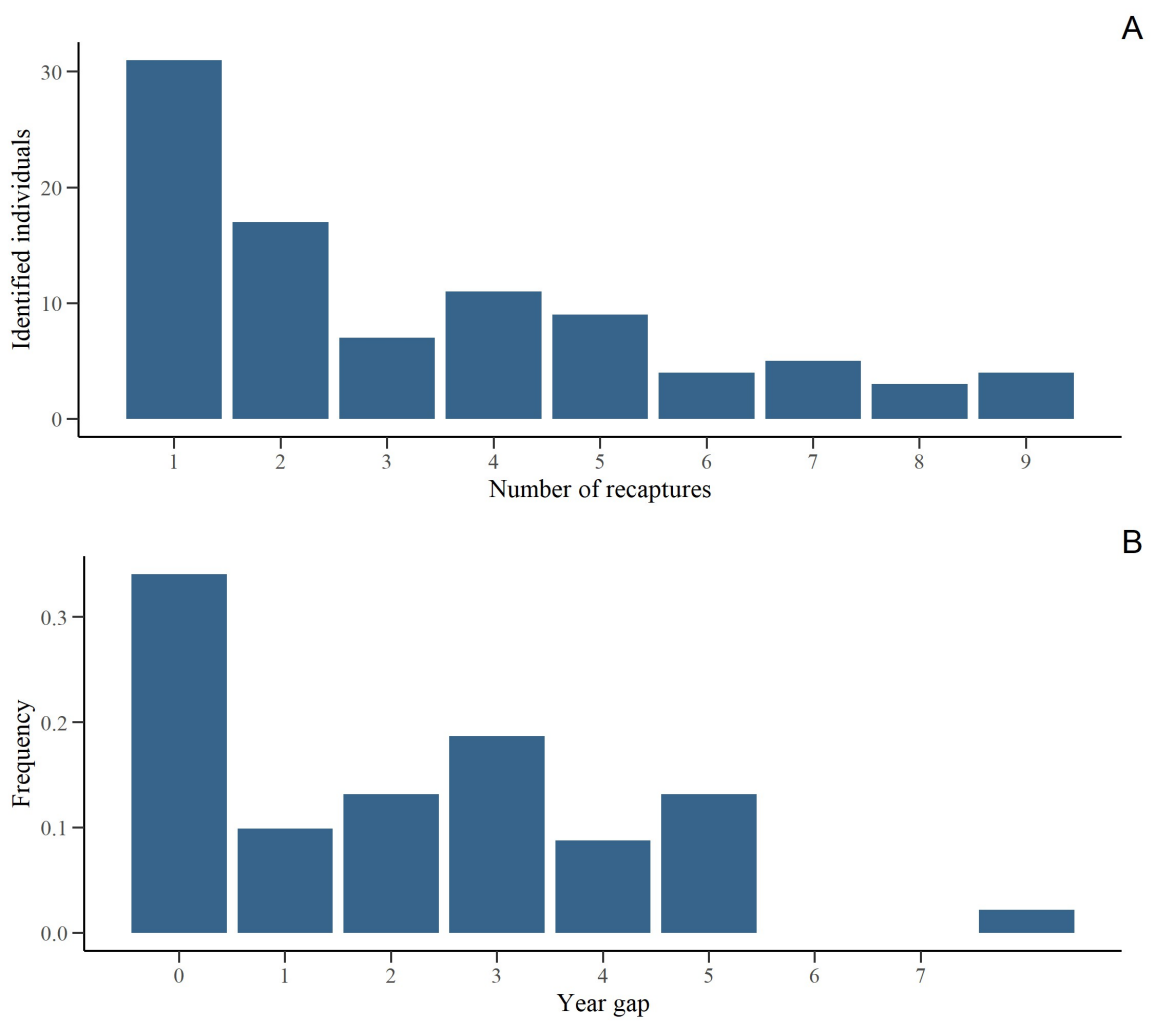
Recaptures and year gaps between recaptures of identified adult dolphins. A) Number of individual adult bottlenose dolphins (*Tursiops* sp.) recaptured between 2012–2021 in southern Port Phillip Bay, south-eastern Australia. **B)** Frequency plot of year gaps between recaptures of individual adult bottlenose dolphins in Port Phillip Bay, south-eastern Australia, between 2012–2021.

**Fig 4:**
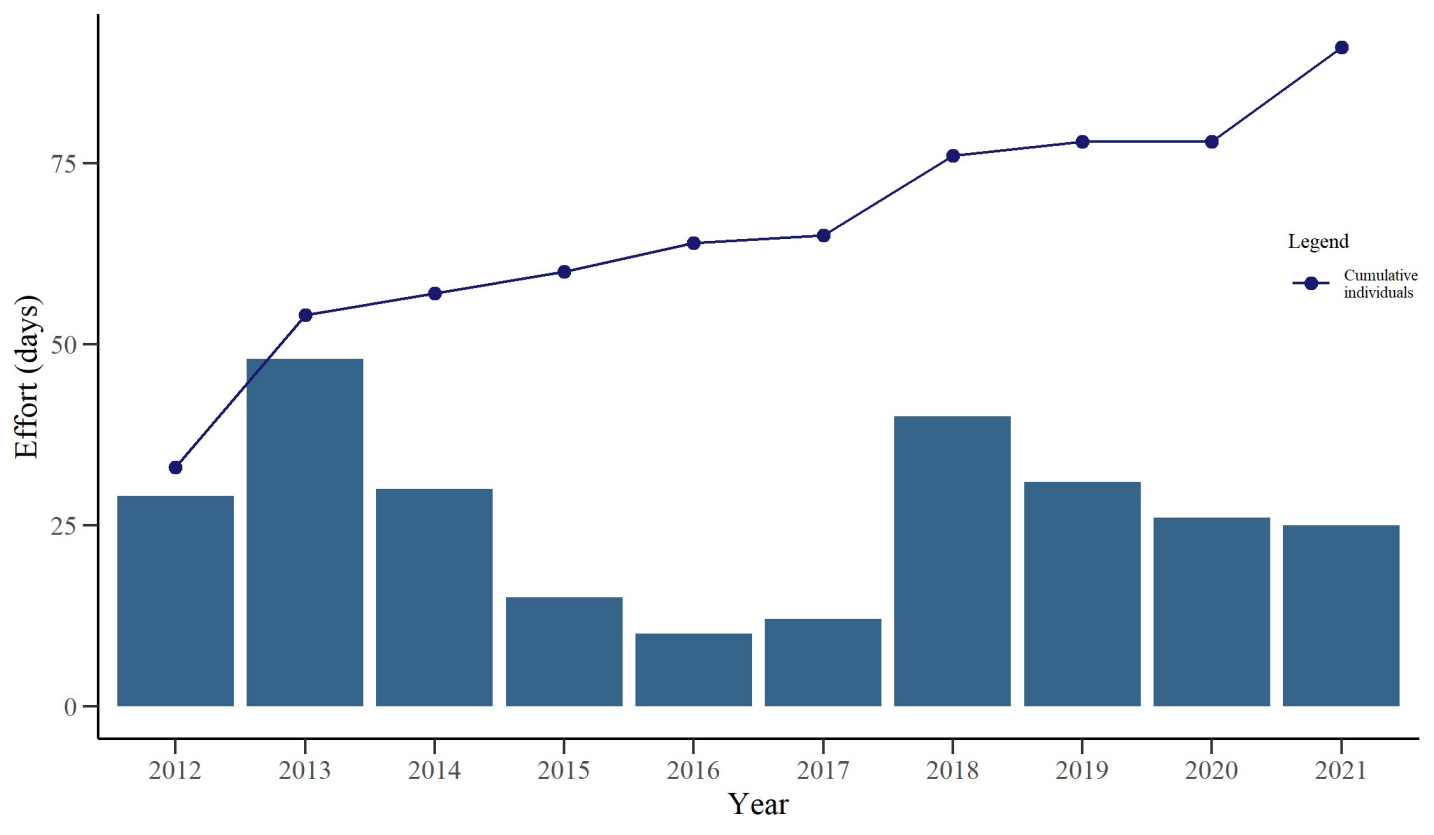
Cumulative plot of newly identified adult bottlenose dolphins (*Tursiops* sp.) in southern Port Phillip Bay, south-eastern Australia. Both the count of newly identified adult dolphins and the effort, as the number of days in which images were available per year between 2012–2021, are shown.

Data collection occurred between 2012–2021, with the start and end dates varying between years (Table 1). A total of 5994 individual digital representations of dolphins were analysed, from which 2517 (42%) were classified as *excellent* and *average* quality. Following analysis within the *Flukebook* portal, a total of 91 adult individuals were identified (Table 2). The number of identified adult individuals per year ranged between 11 and 53 (Fig 2) and was significantly related to the recapture effort as confirmed by the Poisson GLM in accordance with variable distribution (GLM: *z* = 5.99, *P* < 0.001). Of the 91 identified adult individuals, 48 (52%) were seen in only 1 or 2 of the 10 years. Only 4 (4.3%) were observed in at least 9 years (Fig 3A). The gaps between inter-annual recaptures ranged from 1–7 years (Fig 3B). The number of the newly identified adult dolphins per year reached a plateau early in the study (2014), but an increase was observed in both 2018 and 2021 (Fig 4).

**Table 1:** Summary of data collection. Dates and total number of digital images of fins of bottlenose dolphins (*Tursiops* sp.) collected in southern Port Phillip Bay, south-eastern Australia, in each year of the study.

Year	Date range	Fin images (n)
2012	9 Oct—6 Apr	328
2013	26 Oct—17 Apr	694
2014	05 Oct—21 Mar	75
2015	17 Oct—4 Apr	29
2016	14 Oct—24 Apr *	37
2017	13 Oct—15 Mar **	58
2018	11 Nov—24 Apr	357
2019	1 Oct—22 Mar	80
2020	2 Jan—22 Apr	263
2021	17 Nov—28 Apr	4073
**Total**		**5994**

* No data available in December and February

** No data available in February

**Table 2:** Summary of the grading process of the digital images. Count and percentage, of all the collected digital images of bottlenose dolphins (*Tursiops* sp.) in southern Port Phillip Bay, south-eastern Australia, and of the identified ones. The total number of identified adult dolphins and the fin distinctiveness scores are also shown.

Image quality			Identified fins		Identified adult individuals			
*Excellent*	*Average*	*Poor*	*Excellent*	*Average*	Total	D1	D2	D3
1539 (26%)	978 (16%)	3477 (58%)	1204 (78.2%)	761 (77.8%)	91	36	51	4

### Demographic parameters and abundance estimates

Primary and secondary occasions included in the models are summarised in Table 3. The assumption of equal survivability of individuals for the RD models was confirmed by the overall Goodness of Fit of the model to the data (χ^2^ = 33.72, df = 26, *P* = 0.14). The closure assumptions of the secondary occasions were also met (Table 3).

**Table 3:** Summary table of primary and secondary occasions included in the robust design models. These models were used to estimate demographic parameters and population abundance of bottlenose dolphins (*Tursiops* sp.) in southern Port Phillip Bay, south-eastern Australia. The *P* values resulting from both the Stanley and Burnham and the Otis tests for closure are reported.

Primary	Secondary	Stanley&Burnham	Otis
2012	Nov–Dec–Jan–Feb–Mar	0.28	0.02*
2013	Nov–Dec–Jan–Feb–Mar	0.10	2.00
2014	Nov–Dec–Jan–Feb–Mar	0.10	2.00
2015	Nov–Dec–Jan–Feb–Mar	0.80	2.00
2016	Nov–Jan–Mar	0.04*	2.00
2017	Nov–Dec–Jan–Mar	0.25	0.20
2018	Nov–Dec–Jan–Feb–Mar	0.25	0.20
2019	Nov–Dec–Jan–Feb–Mar	0.80	2.00
2020	Jan–Feb–Mar–Apr	0.77	2.00
2021	Nov–Dec–Jan–Feb–Mar	0.05	2.00

* Significant P values

**Fig 5:**
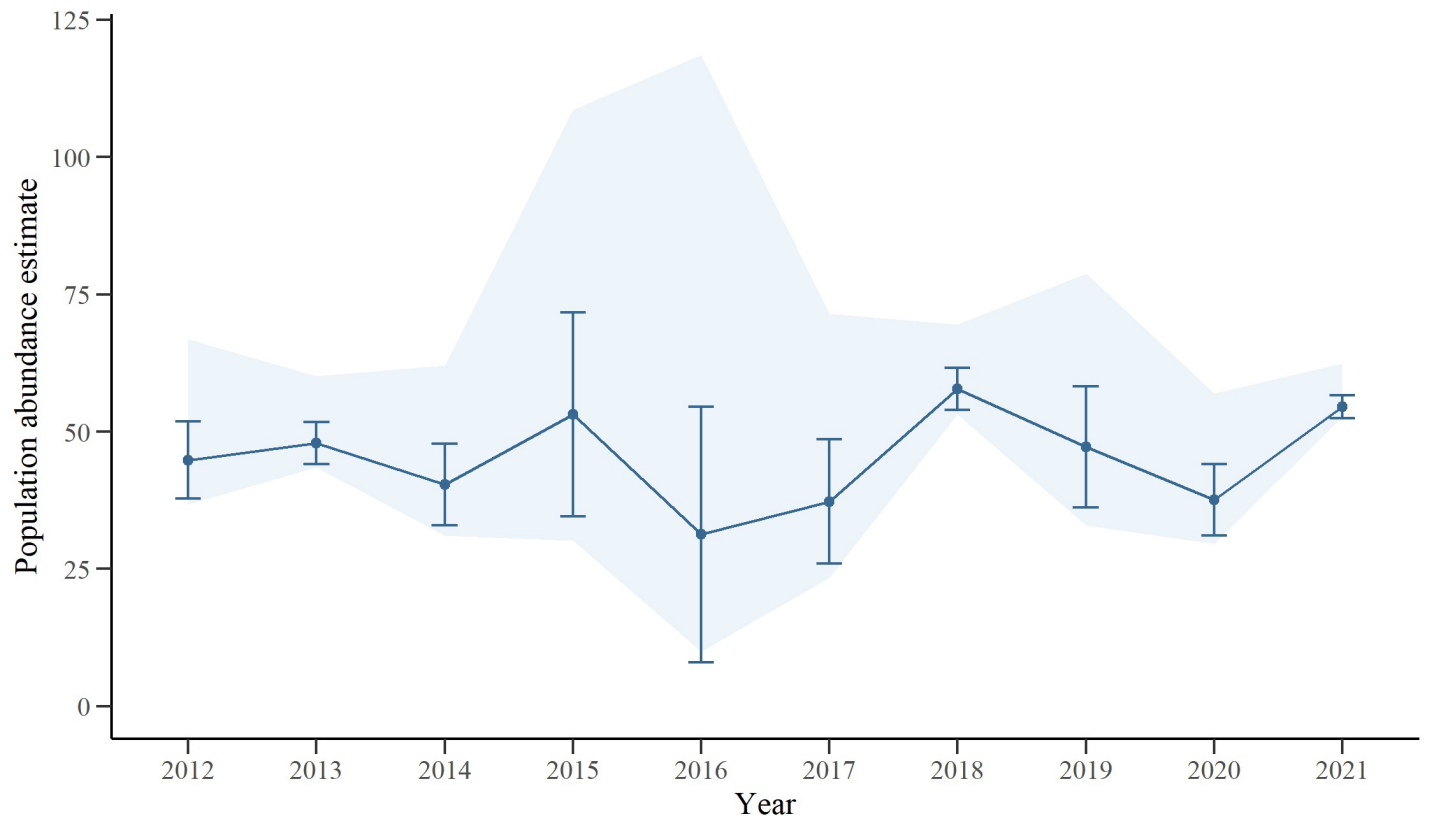
Abundance estimates of marked, adult population. Yearly estimate of the marked adult population abundance of bottlenose dolphins (*Tursiops* sp.) in southern Port Phillip Bay, south-eastern Australia, between 2012–2021. The estimates are not adjusted for unmarked individuals, standard errors are included as error bars and the 95% CI as the shadowed area.

A total of 87 adult individuals were included in the RD models as a result of meeting the CR assumptions by excluding individuals with low fin distinctiveness (D3) and months not included in the previously defined range. Following the AIC selection, the most parsimonious model, out of 56 models, for estimating the demographic parameters of the population included random temporary emigration, constant survival probability, and time- and session-dependent capture probabilities. The inter-annual survival (*S*) of adult individuals was generally high (0.93 ± 0.01, 95% CI: 0.88–0.95), while the random temporary emigration (*Y’* = *Y”*) was low (0.09 ± 0.06, 95% CI: 0.03–0.28). Capture probabilities (*p*) of adult individuals varied greatly between and within primary occasions (years), ranging from 0.02 ± 0.02 (95% CI: 0.003–0.13) to 0.65 ± 0.07 (95% CI: 0.50–0.77). From the parameters obtained, the estimated marked adult population abundance mean in the study area over the decade was 45.2 ± 2.7 individuals (range 31.3–57.8) (Fig 5).

### Social network analysis and reproductive parameters

Only data from 2018–2021 were considered for the social network analysis as these represented the most continuous constant effort. In addition, to reduce potential bias from infrequently sighted individuals, only identified adults seen >5 times throughout this period were included [110, 111]. This reduced data set contained 30 identified adult individuals in 1361 photograph records. The mean HWI was 0.20 ± 0.06, though the most frequent associations were low to moderate (0.10–0.40). Long-term preferred associations were detected (*P* = 0.02) as the standard deviation and coefficient of variance of the real association indices (SD = 0.14, CV = 0.71) were both higher than the random ones (SD = 0.13, CV = 0.67). A similar result was obtained from the temporal analysis as, out of 7 fitted models, the best and most parsimonious one included rapid disassociations and preferred companions to represent the temporal stability of the associations (S1 Table and S1 Fig).

**Fig 6:**
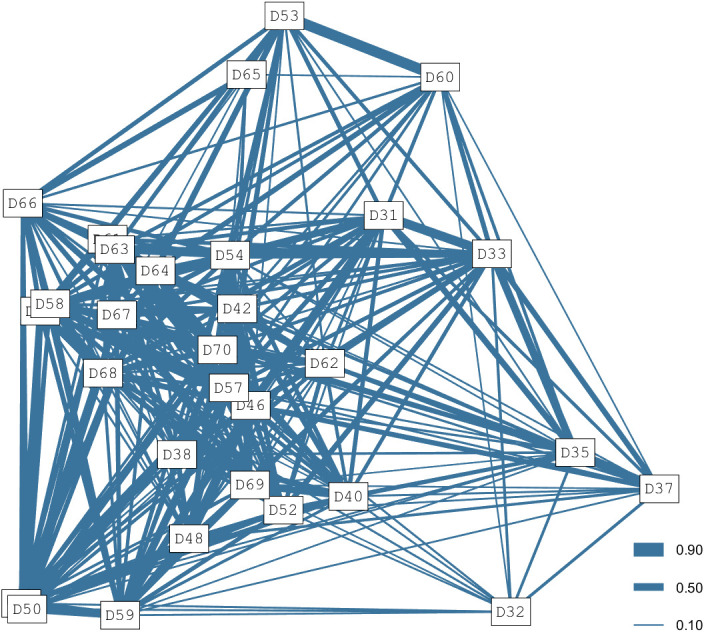
Social diagram of 30 of the marked adult bottlenose dolphins (*Tursiops* sp.) in southern Port Phillip Bay, south-eastern Australia between 2018–2021. The Half-Weight Index values are represented by the thickness of the lines.

The best cluster analysis, based on the AIC, was obtained with the average method. Both the CCC and Q were lower than their thresholds (CCC = 0.70, Q = 0.13). Hence, the resulting division into clusters could not be considered reliable. The lack of division into clusters was also confirmed by the social diagram (Fig 6) and the network metrics (S2 Table) with the dolphins more distant from the central group (D53, D65, D60, D35, D37, D32) presenting network metrics below the mean values.

**Fig 7:**
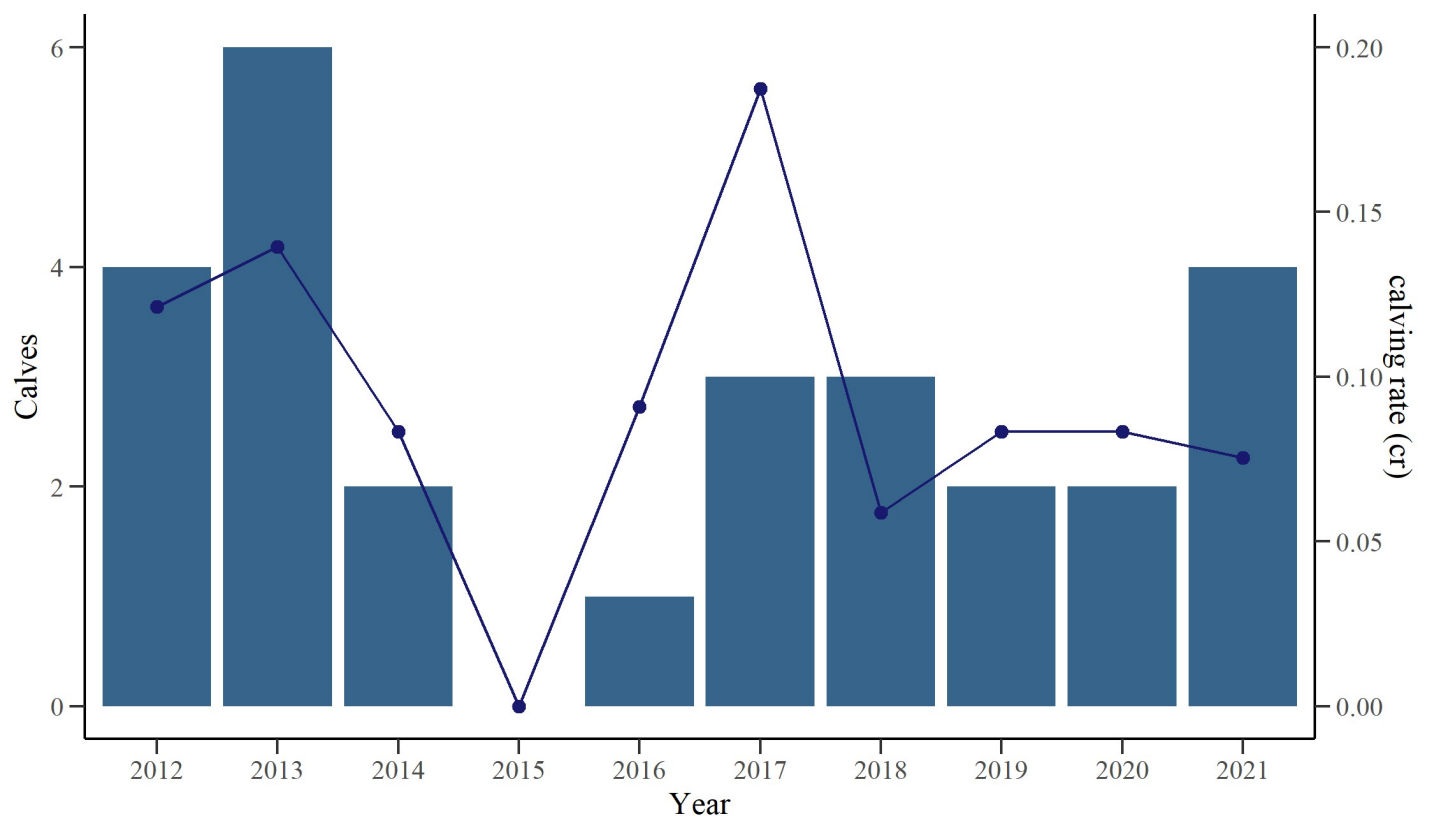
Number of calves (bar graph) and calving rate (new calves per identified individuals per year, line graph) of a population of bottlenose dolphins in southern Port Phillip Bay. Bar plot showing the number of bottlenose dolphin (*Tursiops* sp.) calves born between 2012–2021 and the calculated calving rate (*cr*) in southern Port Phillip Bay, south-eastern Australia.

A total of 27 calves were observed alongside 21 different adults throughout the study period, with the number of new calves observed per year positively related to recapture effort (GLM: *z* = 3.63, *P* < 0.001). The effort-weighted mean number of calves seen per year was 4.0 ± 1.0. The calculated mean IBI across individuals was 3.7 ± 0.8 years, while the *cr* ranged between 0.06–0.19 new calves per identified individuals per year, with the lower rates observed during seasons with lower effort (Fig 7). From the available data, no calves were observed in 2015.

## Discussion

Long-term datasets on life-history metrics are required to assess the population fitness of long-lived species [11]. While the photo-identification techniques provide financially viable, non-invasive means of acquiring such data [31, 116], obtaining it from marine species is still logistically challenging [19, 30]. In the present study, patrons, staff, and volunteers on three ecotourism vessels collected photo-identification images of a poorly studied population of bottlenose dolphins in southern Port Phillip Bay, south-eastern Australia. The effort produced a robust, relatively long-term (10 years) dataset from which abundance, demographic and reproductive parameters, and the social structure of the population were obtained and investigated. The marked adult population abundance estimate was found to be relatively stable throughout the study period, and the life-history and social traits were comparable to those of other bottlenose dolphin populations [117, 118]. These findings suggest that the population is healthy and stable, despite frequent anthropogenic disturbances, and that the data collection methods, made possible only through the involvement of ecotourism operators, are reliable.

### Abundance and demographic parameters

The results of the present study suggest a mean abundance of ca. 45 marked adult bottlenose dolphins over the last decade in southern Port Phillip Bay in a study area of ca. 200 km^2^. While there are no previously published capture recapture abundance estimates for the population in the study area, Hale [59] proposed a population of 80–100 individuals in southern Port Phillip Bay between 1997 and 2002, and Charlton-Robb [119] reported a contemporary effective population size from genetic studies of 81.5 within all of Port Phillip Bay (ca. 1900 km^2^) between 2006–2008. In the present study, the population abundance estimate only refers to the marked adult individuals identified in the southern part of Port Phillip Bay, while accounting for capture probabilities and temporary immigration and emigration.

It was not possible, with the available data, to adjust for the unmarked portion of the population, and to include age classes in the model to estimate the total population abundance. Both these factors result in an underestimation of the total population abundance [100, 105]. However, growth curves of bottlenose dolphins indicate they reach 80% of adult body size at 5–7 years old [102, 120, 121], and population age distributions in Australia [121] and in the Western Atlantic [122] suggest individual >5 years of age comprise between 23–48% of the population. Hence, assuming similar growth parameters and age-distribution, the total population size of bottlenose dolphins in just southern Port Phillip Bay can be estimated at 58–86 individuals.

While caution is needed when comparing dolphin abundances between studies with different approaches and survey methods, it is evident that population or community size of coastal bottlenose dolphins varies greatly throughout the world, from tens to hundreds of individuals [95, 123]. For example, in a study area of ca. 120 km^2^ along the coast of Western Australia, the abundance of bottlenose dolphins was estimated with a RD model at 63–139 individuals and varied with seasonality between 2007–2009 [118]. In Sado Estuary (ca. 200 km^2^), Portugal, a complete count between 2007–2010 resulted in a resident population of 24 individuals [124] while an abundance of 420 individuals was estimated in the Normano-Breton Gulf (ca. 2000 km^2^), France, in 2010 [125] using mark-recapture models for closed populations. Differences in the abundance of dolphins are generally linked to environmental factors, mostly to prey availability and distribution, which affect the carrying capacity of the environment [126, 127]. In southern Port Phillip Bay, the relatively stable estimates of the marked adult population throughout the study period suggest consistently reliable resources for the population. Limited information is currently available on the diet of bottlenose dolphins in Port Phillip Bay but they have been observed to consume garfish (*Hyporhampus* sp.), Gould’s squid (*Nototodarus gouldi*), snapper (*Pagrus auratus*), and barracouta (*Thyrsites atun*) [128]. While the abundance and fine-scale distribution of these species within Port Phillip Bay may change seasonally, their availability has been shown to be relatively stable between years [129, 130].

Previous short-term investigations of the dolphins in southern Port Phillip Bay have observed a decline in sighting success from ecotour vessels and suggested a potential for long-term abandonment of the bay by dolphins due to vessel activity [55]. In addition, whistle frequency was shown to increase in the presence of ecotour boats [84], which could reflect disturbance stress effects [131]. Behavioural changes, such as reduced time spent feeding and increased milling time, were also observed which raised concerns about the long-term effect on energetic intake and expenditure and, hence, overall fitness [63]. While estimates of the population abundance prior to the commencement of ecotour operations are not available, some of the ecotours have been operating in the area for 30+ years and the level of interactions between bottlenose dolphins and tour boats (and other recreational and commercial vessels) is only likely to have increased over time [77]. However, the results of the present study indicate stable abundance estimates over the last decade, suggesting the population is not being detrimentally impacted by the ecotour operations in southern Port Phillip Bay. This is in contrast to Shark Bay, Western Australia, where a long-term study has revealed a ca. 15% decline per km^2^ in the relative population of bottlenose dolphins over 10 years within the area of activity of ecotours [132]. Furthermore, Lusseau et al. [133] have estimated that the threatened population of bottlenose dolphins in Fiordland, New Zealand, could become extinct in a few decades if anthropogenic activities are not reduced.

The best-fitting RD model in the present study resulted in constant survival, time and session-dependent probability of capture, and constant random temporary emigration. The resulting estimates of these parameters are comparable to those of other populations of bottlenose dolphins [134, 135]. The high survival estimate in the present study (0.93), is slightly lower than that observed in populations in the Azores (0.97) [136] and New Zealand (0.92–0.95) [137]. The lower estimate could have been influenced by the presence of younger [105] and transient individuals [138]. Large and slowly reproducing mammals are expected to have high and constant adult survival rates [139]. Hence, the results of the present study suggest the population in Port Phillip Bay is stable.

Random temporary emigration was quite low, with individuals having a 9% probability of not being in the study area. This is generally lower than in previous studies where random temporary emigration has been found (i.e. 16% probability in [100] and between 33–66% in [140]. Temporary emigrations of bottlenose dolphins could be related to differences in site fidelity and home ranges between sexes, or to the distribution and availability of food resources [138, 141, 142]. In addition, the low estimate in comparison to other studies could reflect an influence of heterogeneity in capture probabilities [136]. Indeed, the latter varied greatly between sessions which is most likely due to changes in effort, with lower effort resulting in lower capture probability estimates.

During summer 2022–2023 three adult dolphins frequently identified in Port Phillip Bay were observed in Cat Bay (38.5018° S, 145.1296° E) by the staff of *Wildlife Coast Cruises*, an ecotour operator at Phillip Island 40 km from the study area (Sue Mason, pers. comm.).

Two young dolphins were in association with the three adult dolphins suggesting the presence of females within the group. These findings demonstrate that individuals of the study population do move outside Port Phillip Bay at times. Hence, future photo-identification surveys should be planned in a wider area to better understand the population’s home range. These movements should also be considered in future population abundance models to obtain a more precise estimate [143].

### Social structure and reproductive parameters

Like in many congenerics, the population of bottlenose dolphins within the present study showed weak associations typical of a fission-fusion social structure [144]. Generally, the social structure of a population is driven by the need to maximize fitness according to the ecological conditions, mostly to prey availability and distribution, and to predation risk [13, 145]. In enclosed areas, where food resources are available and relatively predictable, such as in Port Phillip Bay [79], dolphins should form small groups with loose bonds [13]. Hence, long-term changes in association strengths may reveal underlying ecosystem changes in Port Phillip Bay. For example, in southern Moreton Bay (Queensland, Australia), the social structure of bottlenose dolphins changed as a consequence of a reduction in trawling effort [146].

Some moderate to strong associations were observed in the present study, as well as some preferred long-term companions, which was also supported by the temporal stability of the associations suggesting rapid disassociations and preferred companions. The presence of such bonds in bottlenose dolphin communities has been observed as being sex-specific or driven by the need for a high level of cooperation due to environmental factors [43, 147–150].

Social clusters are commonly found in populations of bottlenose dolphins [151, 152] and they are driven by many different factors, such as feeding strategies, habitat use, age, and sex [42, 146, 151–154]. Analysis of associations in the present study did not reveal cluster division but some individuals had higher social network statistics scores, suggesting a more central role in the community. However, evidence of the presence of possible clusters within Port Phillip Bay was obtained during three days of sampling in 2022 ca. 30 km north-west of the study area near Corio Bay (38.1167° S, 144.4333° E) (Fig 1). While not included in the current study, these surveys demonstrated the presence of at least six identifiable dolphins not previously recorded in the study area associating with a few individuals identified as part of the present study. These findings support the temporary emigration parameter obtained in the RD model and suggest the presence of multiple interacting clusters within different areas of Port Phillip Bay. Such a scenario would infer a potentially much larger population of bottlenose dolphins in Port Phillip Bay than previously assumed [59, 119].

The reproductive parameters obtained from the photo-identification analysis are comparable to those of other bottlenose dolphin populations. The observed calving rate (maximum of 0.19) changed with effort, indicating it was stable throughout the study period. A similar result was observed in the Moray Firth (Scotland), where the calving rate varied between 0.05–0.21 [115], while lower rates were observed in Doubtful Sound (New Zealand, 0.04) [117] and Shannon Estuary (Ireland, 0.072) [101] populations. Despite the low number of females seen with calves multiple times over the study period, an IBI estimate of 3.7 years could still be obtained. Similar IBI values have been recorded in much larger studies in Port River estuary, Australia (3.8 years) [155], Scotland (3.3 years) [115] and in New Zealand (2.1–5.3) [117].

Reproductive parameters reflect the impacts of environmental and anthropogenic pressures affecting the population [114]. Due to the high energetic cost of reproduction [156–158], calving rate could change in response to long-term variations in environmental conditions [159, 160]. While the IBI could become longer if the environmental conditions worsen [161], it is observed to become shorter when calves die in early post-partum [114]. The similarity in reproductive parameters observed in the present study to that in other bottlenose dolphin populations, and the apparent stability of the estimated abundance over the last decade, suggest neither environmental nor anthropogenic pressures are significantly negatively impacting the southern Port Phillip Bay population.

The present study was only possible through the data collection efforts of ecotourism patrons, staff, and volunteers. The results suggest the population of bottlenose dolphins in southern Port Phillip Bay has remained stable over the last decade, with ca. 45 marked adult individuals (with a potential total population size of >80 individuals). The evidence of associations between unknown and known dolphins in other areas of the Port Phillip Bay (Corio Bay) suggests a larger total population in the whole of the bay. In addition, the demographic, reproductive and social structure parameters are comparable to those of many other bottlenose dolphin populations. These findings further highlight the importance of collecting and analysing long-term data sets to properly assess life-history traits of social species. Furthermore, with the financial and logistical difficulties involved in the collection of such data sets, this study further highlights the benefit of collaborations with ecotour operators for obtaining such vital information on cetacean populations in Port Phillip Bay and worldwide.

The possible presence of different clusters of bottlenose dolphins outside the study area indicates sampling effort should be extended to adjacent areas within, as well as outside, Port Phillip Bay. In addition, surveying at other times of the year may reveal seasonality in population abundance. A greater sampling effort and accuracy are required to enhance the reliability of estimates, especially by including home range, age, and sex in the models, further emphasising the importance of continued collaborative arrangements with and support from tour operators. Since a proper collaboration only started in the last few years, the data available before the introduction of the protocol was collected opportunistically. Now, that the importance of this collaboration is clear, a more standardised data collection protocol can be developed, better data obtained, and incorporated to the previous.

## Supporting information

S1 Fig Temporally lagged association rates for 30 adult bottlenose dolphins in Port Phillip Bay, south-eastern Australia. Both the Lagged and Null association rates are included.
(TIF)

S1 Table Summary table of the lagged association rate models. Models for the temporal analysis of the 30 adult bottlenose dolphins (*Tursiops* sp.) in southern Port Phillip Bay, south-eastern Australia between 2018–2021.
(PDF)

S2 Table Network measures of 30 adult bottlenose dolphins (*Tursiops* sp.) in southern Port Phillip Bay, south-eastern Australia between 2018–2022. The standard deviations of each measure is shown in parenthesis and the standard errors of the mean were calculated with a Bootstrap of 1000 replicates (in square brackets).
(PDF)
