# Supplementary figures and images for: Using ecotourism boats for estimating the abundance of a bottlenose dolphin population in south-eastern Australia
Source: PLoS One. 2023 Aug 4;18(8):e0289592. doi: 10.1371/journal.pone.0289592 (PMC10403133; doi:10.1371/journal.pone.0289592)

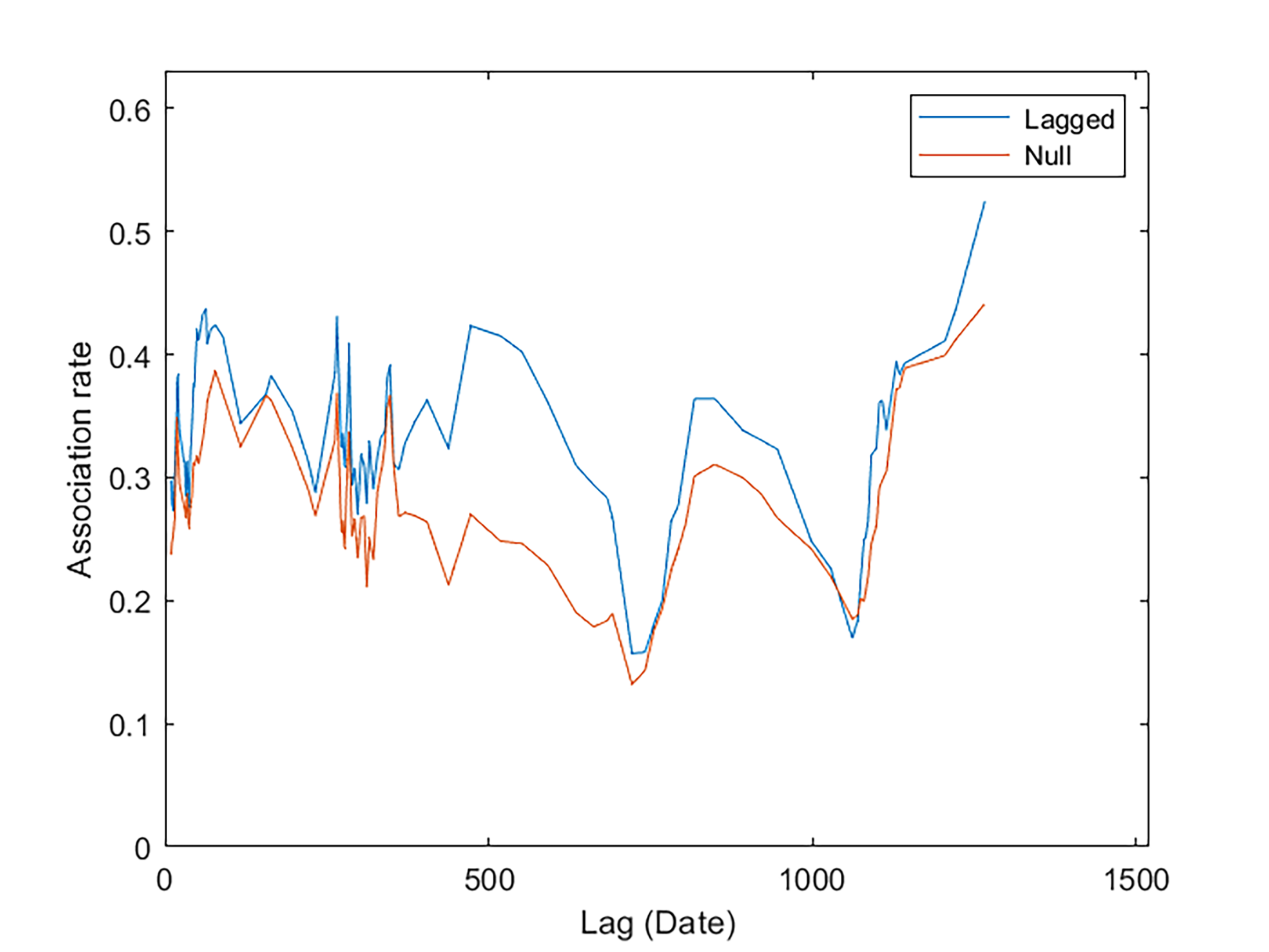

Supplement: S1 Fig — Both the Lagged and Null association rates are included. (TIF) [file pone.0289592.s001.tif]
