# Supplementary material for: Using ecotourism boats for estimating the abundance of a bottlenose dolphin population in south-eastern Australia
Source: PLoS One. 2023 Aug 4;18(8):e0289592. doi: 10.1371/journal.pone.0289592 (PMC10403133; doi:10.1371/journal.pone.0289592)
Supplement: S1 Table — Models for the temporal analysis of the 30 adult bottlenose dolphins (Tursiops sp.) in southern Port Phillip Bay, south-eastern Australia between 2018–2021. (PDF) [file pone.0289592.s002.pdf]

Model	Formula	N of parameters	Estimates of parameters	QAIC
Closed units	1	0		45463.4489
Rapid dissociation and preferred companions	a1	1	a1=0.27939	2029.2496
Casual acquaintances	exp(-a1*td)	1	a1=0.0029282	3756.225
**Rapid dissociation and casual acquaintances**	**a2exp(-a1*td)**	**2**	**a1=-4.4145e-05**<br>**a2=0.2736**	**2031.0291**
Rapid dissociation and preferred companions and casual acquaintances	a2+a3*exp(-a1*td)	3	a1=9.0265<br>a2=0.28043<br>a3=-961.6312	2032.1782
Preferred companions and casual acquaintances	a2+(1-a2)*exp(-a1*td)	2	a1=31.1708<br>a2=0.27944	2031.2496
Two levels of casual acquaintances	a3*exp(-a1*td)+(1-a3)*exp(-a2*td)	3	a1=0.72416<br>a2=-7.7253e-05<br>a3=0.73406	2046.2065
Rapid disassociation and two levels of casual acquaintances	a3*exp(-a1*td)+a4*exp(-a2*td)	4	a1=34.8877<br>a2=-4.4002e-05<br>a3=33.5248<br>a4=0.27357	2035.0291
