# Supplementary material for: Using ecotourism boats for estimating the abundance of a bottlenose dolphin population in south-eastern Australia
Source: PLoS One. 2023 Aug 4;18(8):e0289592. doi: 10.1371/journal.pone.0289592 (PMC10403133; doi:10.1371/journal.pone.0289592)
Supplement: S2 Table — The standard deviations of each measure is shown in parenthesis and the standard errors of the mean were calculated with a Bootstrap of 1000 replicates (in square brackets). (PDF) [file pone.0289592.s003.pdf]

ID	Strength	Eigenvector centrality	Reach	Clustering coefficint	Affinity
D31	6.26[1.86]	0.19[0.05]	39.74[16.68]	0.27[0.06]	6.35[0.91]
D32	1.81[0.87]	0.05[0.03]	10.46[4.80]	0.24[0.10]	5.77[0.95]
D33	6.80[1.76]	0.19[0.06]	40.39[14.98]	0.24[0.05]	5.94[0.91]
D35	4.61[1.22]	0.13[0.05]	26.98[9.50]	0.24[0.06]	5.85[0.94]
D37	3.36[1.13]	0.09[0.04]	19.93[8.61]	0.26[0.08]	5.93[1.04]
D38	4.62[1.28]	0.14[0.05]	29.66[9.94]	0.27[0.06]	6.42[0.89]
D39	6.75[1.75]	0.21[0.05]	43.67[17.53]	0.29[0.05]	6.47[1.00]
D40	6.00[1.63]	0.18[0.05]	36.76[13.52]	0.25[0.05]	6.13[0.86]
D42	6.88[1.61]	0.21[0.04]	42.86[14.96]	0.26[0.04]	6.23[0.85]
D46	7.50[1.77]	0.23[0.05]	46.64[16.26]	0.26[0.05]	6.22[0.91]
D48	4.74[1.53]	0.15[0.05]	31.05[12.99]	0.29[0.09]	6.55[0.95]
D49	6.03[1.26]	0.19[0.05]	38.10[12.57]	0.28[0.05]	6.32[0.96]
D50	5.93[1.25]	0.18[0.05]	37.78[12.55]	0.29[0.05]	6.37[0.96]
D52	6.34[1.60]	0.19[0.04]	39.50[14.42]	0.26[0.05]	6.23[0.95]
D53	4.91[1.39]	0.15[0.05]	30.43[12.24]	0.26[0.06]	6.19[1.03]
D54	7.84[1.64]	0.24[0.03]	48.91[16.20]	0.26[0.05]	6.24[0.90]
D57	7.90[1.61]	0.24[0.04]	48.40[15.65]	0.25[0.05]	6.13[0.90]
D58	6.54[1.69]	0.21[0.04]	42.41[17.23]	0.28[0.06]	6.49[1.04]
D59	6.36[1.49]	0.20[0.05]	40.39[14.00]	0.28[0.05]	6.35[0.94]
D60	4.48[1.33]	0.13[0.05]	27.17[11.33]	0.26[0.06]	6.06[1.02]
D61	4.08[1.39]	0.13[0.04]	27.22[11.55]	0.30[0.06]	6.68[0.91]
D62	5.55[1.26]	0.16[0.04]	33.82[11.24]	0.24[0.04]	6.09[0.88]
D63	8.04[1.22]	0.24[0.03]	49.91[14.06]	0.25[0.04]	6.21[0.89]
D64	6.95[1.30]	0.21[0.04]	42.94[13.21]	0.26[0.05]	6.18[0.89]
D65	1.42[0.80]	0.04[0.03]	9.16[5.65]	0.32[0.11]	6.45[1.18]
D66	5.96[1.59]	0.18[0.05]	36.88[14.83]	0.26[0.05]	6.18[0.98]
D67	6.64[1.39]	0.20[0.04]	42.00[13.67]	0.26[0.05]	6.32[0.90]
D68	6.22[1.76]	0.20[0.04]	40.08[17.03]	0.28[0.06]	6.44[1.02]
D69	6.36[1.52]	0.19[0.04]	40.05[14.61]	0.26[0.05]	6.29[0.92]
D70	7.42[1.25]	0.22[0.02]	46.38[13.96]	0.26[0.04]	6.25[0.89]
Overall means	5.81(1.63) [0.76]	0.18(0.05) [0.00]	36.32(10.23) [9.72]	0.27(0.02) [0.04]	6.24(0.21) [0.86]
